# Understanding the Barriers to Pooled SARS-CoV-2 Testing in the United States

**DOI:** 10.1128/spectrum.00312-21

**Published:** 2021-08-11

**Authors:** Eli P. Fenichel, R. Tobias Koch, Anna Gilbert, Gregg Gonsalves, Anne L. Wyllie

**Affiliations:** a Yale School of the Environment, New Haven, Connecticut, USA; b Department of Epidemiology of Microbial Diseases, Yale School of Public Health, New Haven, Connecticut, USA; c Department of Mathematics, Yale University, New Haven, Connecticut, USA; d Department of Statistics & Data Science, Yale University, New Haven, Connecticut, USA; Keck School of Medicine of the University of Southern California

**Keywords:** SARS-CoV-2, covid, diagnostics, infectious disease, pooled testing, pooling, screening, surveillance

## Abstract

Pooled testing for severe acute respiratory syndrome coronavirus 2 (SARS-CoV-2) detection is instrumental for increasing test capacity while decreasing test cost. Pooled testing programs permit sustainable, long-term surveillance measures, which are essential for the early detection of virus resurgence in communities or the emergence of variants of concern. While numerous pooled approaches have been proposed to increase test capacity, uptake by laboratories has been limited. On 9 December 2020, we invited 362 U.S. laboratories that inquired about the Yale School of Public Health SalivaDirect test to participate in a survey to evaluate testing constraints and pooling strategies for SARS-CoV-2 testing. The survey was distributed using Qualtrics, and three reminders were sent. The survey closed on 21 January 2021. Of 93 responses received (25.7% response rate), 90 were from Clinical Laboratory Improvement Amendments (CLIA)-certified laboratories conducting SARS-CoV-2 testing. The remaining three were excluded from the analyses. Responses indicated that the major barriers to the uptake of pooled testing in the United States may not simply be the number of tests a laboratory can process per day, but rather the lack of clear protocols and adequate resources; laboratories are working with fixed physical and human capital constraints. Importantly, laboratories across the country are heterogeneous in infrastructure and workflow. The need for SARS-CoV-2 testing will remain for years to come. Testing programs can be maintained through pooled PCR testing strategies, and while statisticians, operations researchers, and others with expertise in sampling design have important value to add, laboratories require support on how to transition from traditional diagnostic testing to pooled surveillance.

**IMPORTANCE** While numerous pooled SARS-CoV-2 testing approaches have been described in an effort to increase testing capacity and decrease test prices, uptake by laboratories has been limited. Responses to our survey of United States-based laboratories highlight the importance of consulting end-users—those that solutions are being designed for—so challenges can be addressed in a manner tailored to meet the specific needs out in the field. It may be surprising to those designing pooled testing strategies to learn that laboratories view pooling as more time-consuming than testing samples individually, and therefore that it is thought to create delays in test reporting.

## INTRODUCTION

Early in the pandemic, testing for severe acute respiratory syndrome coronavirus 2 (SARS-CoV-2) emerged as an Achilles’ heel of the U.S. national response (1). The failure to scale up testing programs rapidly and delays in test processing and return of results led to delays downstream in self-isolation, diagnosis, and treatment, undercounting of infections, fear, and confusion. Proposals for novel strategies involved pooling of samples from multiple individuals into one testing run. Many countries, including South Korea (2), Israel, Germany, South Africa, and China (3), rapidly implemented pooling as part of national plans. The United States never implemented pooling as part of its own strategy in earnest (4). According to Google Scholar, over 10,000 papers with the key words “COVID” and “pooling” were “published” in 2020. Pooling samples to increase throughput is not a new idea (5). For most realistic test-positive levels, even simple pooling designs greatly increase capacity (6). So, why has pooled testing been so rare in the United States? To resolve this question, we explored the barriers facing laboratories to expand testing capacity.

## RESULTS

Of the 362 United States-based laboratories invited to participate in our survey to evaluate testing constraints and pooling strategies for SARS-CoV-2 testing, 93 responses were received (25.7% response rate; locations shown in the supplemental material). Of these, 90 responses were from Clinical Laboratory Improvement Amendments (CLIA)-certified laboratories conducting SARS-CoV-2 testing. The remaining three were excluded from the analyses. Nearly half of the laboratories responding were for profit (*n* = 42), followed by university affiliated laboratories (*n* = 9), with the remainder made up of community, nonprofit, and government laboratories. The respondent laboratories reported serving a variety of testing populations, with the bulk consisting of student (*n* = 44), outpatient (*n* = 47), and community testing (*n* = 58); only 29 laboratories reported inpatient testing. While most conducted diagnostic testing, many laboratories also reported testing for general surveillance (*n* = 41) and screening for specific events (*n* = 37). The high reporting of diagnostic testing, which is likely representative across all CLIA laboratories, is important in the context of pooling, which differs from testing for screening and surveillance at the population scale.

The survey included an open-ended question about the barriers to pooling. A common major barrier to the implementation of pooling by laboratories was a lack of methods accepted by the necessary authorities, including the FDA, CLIA regulations, or their own laboratory directors (*n* = 34). Reports of a lack of clear open-source protocols and guidance on the methodology for implementing pooled testing using existing infrastructure contributed to this (*n* = 14). Many laboratories expressed that even if protocols were available, a lack of time and resources limited their ability to validate these in house, preventing proof-of-concept implementation (*n* = 17). Thus, demonstration of the effectiveness of pooling in their setting is lacking. In that regard, laboratories also reported concerns that local case positivity rates were simply too high to warrant pooling (*n* = 10), despite recent lab-based data demonstrating the benefit of pooling five samples up to an ∼30% test-positive rate (6). Throughout, there were a substantial number of concerns about the effect of pooling on the sensitivity of detection and the increased risk for false-negative results due to sample dilution (*n* = 14).

Operational and administrative barriers were also a common theme. Laboratories reported that reflex testing of the most common Dorfman approach (5), in which samples from positive pools are revisited for individual retesting, are too logistically difficult to manage or are too resource demanding (*n* = 6). Laboratories viewed the need to revisit samples of positive pools as disruptive to the standard testing flow and staffing practices, expressing concerns that this would add to the time of reporting out positive tests (*n* = 14). A major concern was the additional logistics required for following samples through the pooled testing process and the lack of the necessary software to track samples through the workflow (*n* = 9). Important to any testing program were concerns regarding reimbursement and billing when samples are tested in pools, as well as contract obligations (*n* = 3). Limited staff to dedicate to tackling these issues came up repeatedly (*n* = 16).

Survey responses demonstrated that the SARS-CoV-2 testing environment in the United States is highly heterogeneous, particularly in regard to the supplies, the testing platforms, and the variety of sample types received. While a majority of laboratories use 96-well PCR instruments (*n* = 39), 384-well instruments are also common (*n* = 11), with some laboratories reporting nonstandard plate sizes in upstream processes (*n* = 24). Additionally, some laboratories utilize multiple instruments in their testing procedures (*n* = 12), which can differ in the number of samples each can process. These interlaboratory variations in testing workflows are important for pooled testing strategies; a protocol developed for one laboratory may not be easily translated into another. Those developing pooled testing strategies need to recognize that laboratories are working with fixed physical and human capital constraints and that new laboratories are not being built around pooling strategies; such constraints cannot be assumed away.

**FIG 1:**
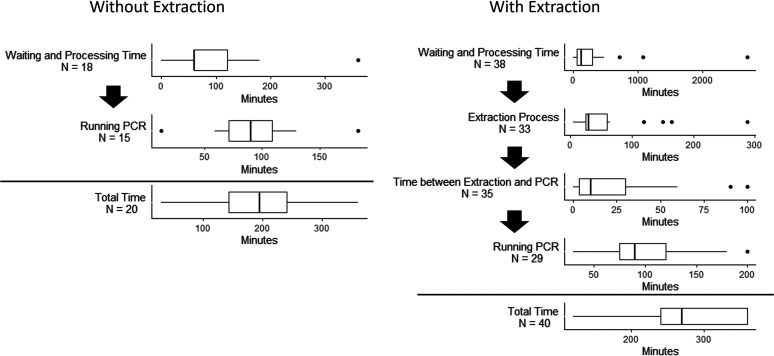
The SARS-CoV-2 testing environment in the United States is highly heterogeneous. Responses to our survey indicate substantial variation in SARS-CoV-2 testing workflows in laboratories throughout the United States. Depicted is the variation in timing constraints for critical steps of SARS-CoV-2 testing workflows with or without RNA extraction.

Higher test throughput, lower costs per test, and faster turnaround times are all margins for testing improvement. Increasing the number of samples per test in a pooling structure can be part of that solution. Laboratories using an extraction process reported an average of 4.6 h to test a sample from start to finish (time without extraction = 3.0 h), although substantial variation exists (Fig. 1). The small number of laboratories (*n* = 6) in our sample that engage in pooling reported total testing time in line with these figures. On average, samples wait in laboratories for 93 min prior to workflows without extraction and for 279 min prior to extraction-based tests. The average time to set up and run PCR is 94 min, with little difference between laboratories. For laboratories performing full RNA extraction, this process takes an average of 57 min. Reset or cycle times between processes are on average 18 min for extraction and PCR. These times are one motivation for laboratories focused on faster turnaround to be wary of designs that require retesting of samples, which can add additional hours to the process.

## DISCUSSION

The need for testing will remain for the years to come. Pooled testing offers sustainable surveillance measures that support long-term programs, which are essential for the early detection of virus resurgence or the emergence of variants of concern. While testing programs can be maintained by pooled PCR testing, laboratories require guidance on how to transition from traditional diagnostic testing to pooled surveillance. Importantly, our survey suggests that the major barriers to uptake and implementation of pooled testing in the United States may not simply be the number of tests a laboratory can process per day, but rather the lack of adequate resources and guidance on clinical best practices to transition to pooling. Additionally, a number of laboratories did not feel that pooled testing was necessary given current testing capacity constraints at present. If the appropriate resources were made available, however, laboratories reported that even with current nonpooled testing strategies, they could on average, increase testing capacity by 60%. While laboratories need to see more evidence supporting the ability of pooled testing strategies to successfully move from theory to a reliable and resource-saving laboratory practice, logistical solutions to support implementation and general processing remain vital. Responses to our survey highlight the importance of consulting end users. While statisticians, operations researchers, and others with expertise in sampling design have important value to add, end users will often identify real-world constraints that must be accounted for during design; statisticians and operations researchers often appear unaware of these constraints. This suggests that there are still substantial opportunities for these communities to collaborate and enhance testing capacity, as it may be surprising to those designing pooling strategies to learn that laboratories view pooling as more time consuming, delaying test reporting.

## MATERIALS AND METHODS

On 9 December 2020, we invited 362 laboratories that had contacted the Yale School of Public Health and expressed interest in implementing the SalivaDirect test (7) to participate in a survey to evaluate testing constraints and pooling strategies for SARS-CoV-2 testing. The survey was distributed using Qualtrics, and three reminders were sent. The survey closed on 21 January 2021. Laboratories were surveyed about their geographic location, laboratory type, populations tested, SARS-CoV-2 testing (capacity and test turnaround time per sample type), laboratory workflows, and an open-ended question regarding the barriers to pooled testing.
