# Supplementary material for: Understanding the Barriers to Pooled SARS-CoV-2 Testing in the United States
Source: Microbiol Spectr. 2021 Aug 11;9(1):10.1128/spectrum.00312-21. doi: 10.1128/spectrum.00312-21 (PMC8552767; doi:10.1128/spectrum.00312-21)
Supplement: SUPPLEMENTAL FILE 1 — Supplemental material. Download SPECTRUM00312-21_Supp_1_seq3.pdf, PDF file, 0.1 MB [file spectrum00312-21_supp_1_seq3.pdf]

SUPPLEMENTARY FIGURE

Understanding the barriers to pooled SARS-CoV-2 testing in the United States

Eli P. Fenichel, Ph.D[a], R. Tobias Koch, B.Sc[b], Anna Gilbert, Ph.D[c,d], Gregg Gonsalves, Ph.D[b], and Anne L. Wyllie, Ph.D[b#]

[a] Yale School of the Environment, 195 Prospect St. New Haven, CT, 06511

[b] Department of Epidemiology of Microbial Diseases, Yale School of Public Health, 60 College St, New Haven, CT 06510

[c] Department of Mathematics, Yale University, 10 Hillhouse Avenue, New Haven, CT, 06511,

[d] Department of Statistics & Data Science, Yale University, 10 Hillhouse Avenue, New Haven, CT, 06511

[#]Corresponding author: Anne Wyllie, anne.wyllie@yale.edu, 203-781-6942

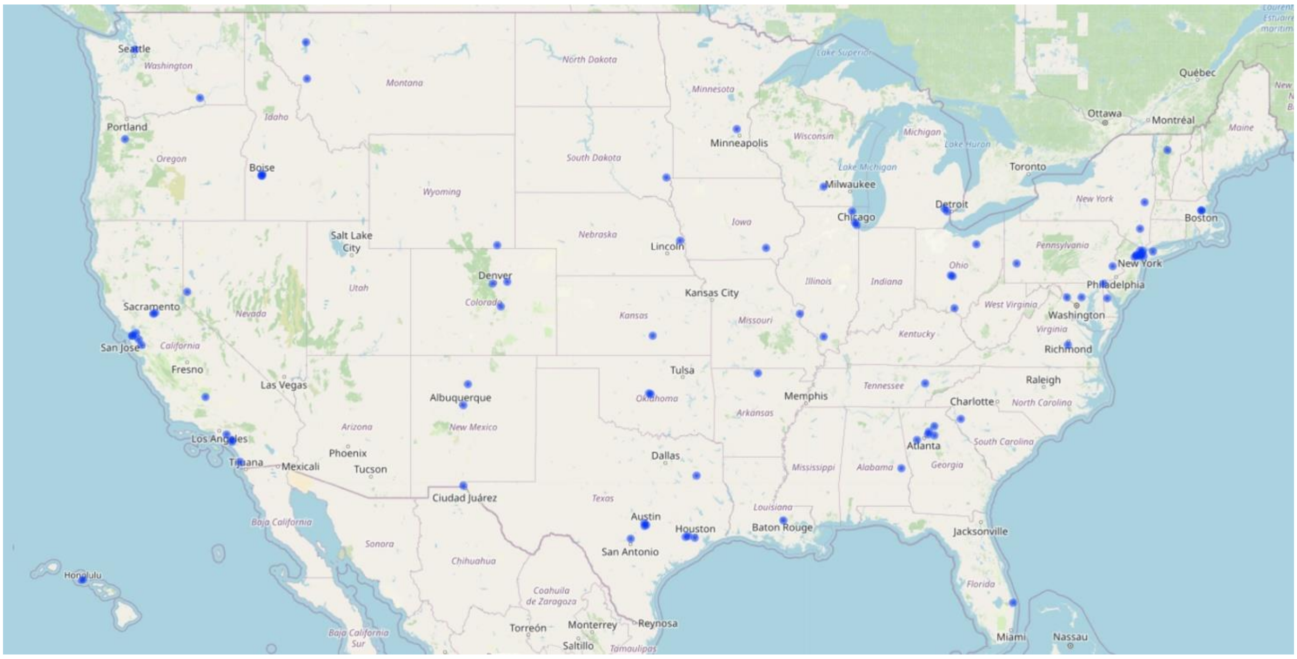

Supplementary Figure. Geographic locations of laboratories surveyed regarding barriers to pooled testing in the US. Of the 362 US-based laboratories invited to participate in our survey to evaluate testing constraints and pooling strategies for SARS-CoV-2 testing, 93 responses were received (25.7% response rate). Blue dots indicate the geographical location of the respondent laboratories.
